# Supplementary figures and images for: Cell-Type Specific Analysis of Selenium-Related Genes in Brain
Source: Antioxidants (Basel). 2019 May 5;8(5):120. doi: 10.3390/antiox8050120 (PMC6562762; doi:10.3390/antiox8050120)

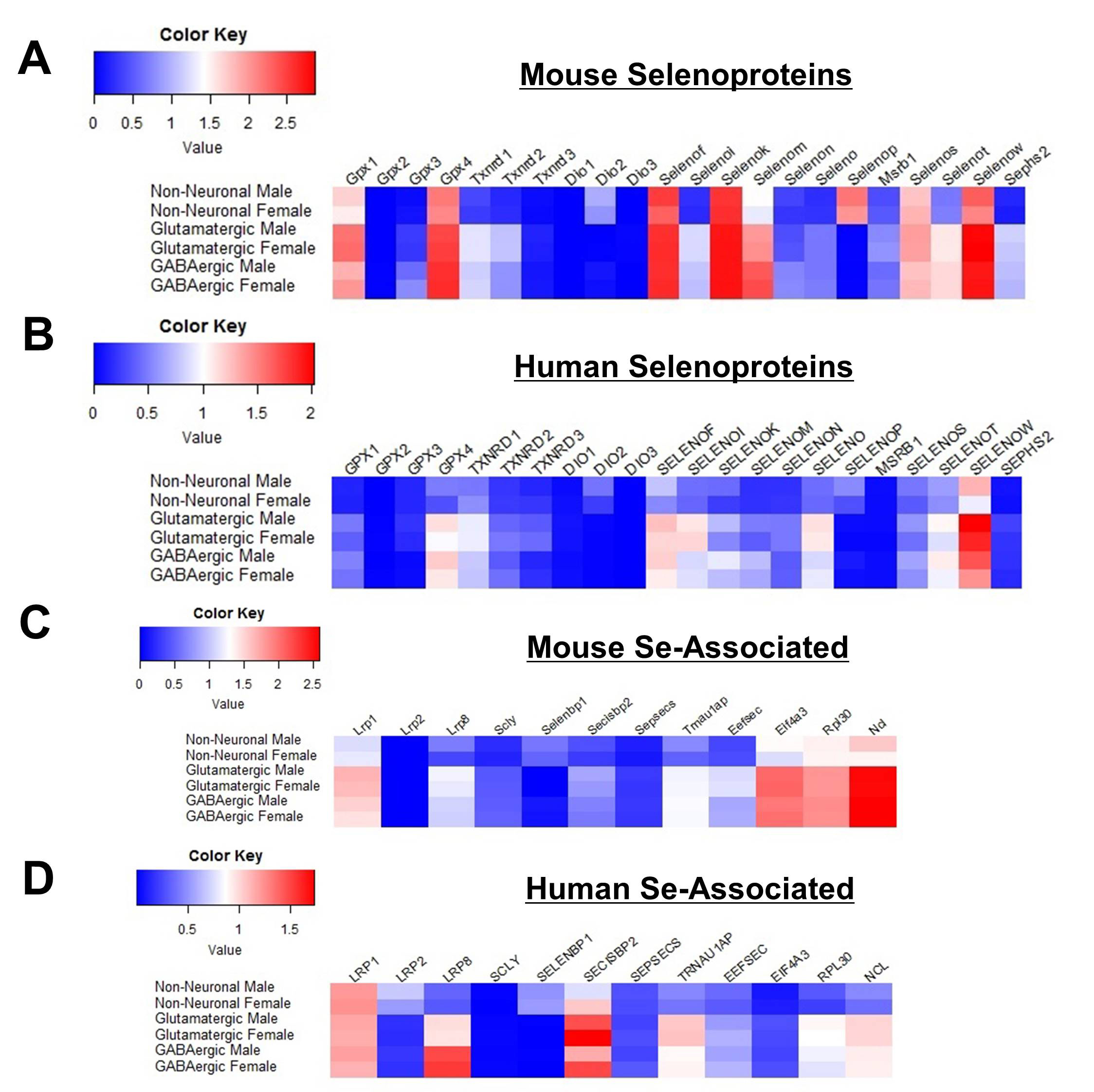

Supplement: Supplementary file 1 [file antioxidants-08-00120-s001.zip › supple/Supplemental Figure.jpg]
